# Both Ubiquitin Ligases FBXW8 and PARK2 Are Sequestrated into Insolubility by ATXN2 PolyQ Expansions, but Only FBXW8 Expression Is Dysregulated

**DOI:** 10.1371/journal.pone.0121089

**Published:** 2015-03-19

**Authors:** Melanie Vanessa Halbach, Tanja Stehning, Ewa Damrath, Marina Jendrach, Nesli Ece Şen, A. Nazlı Başak, Georg Auburger

**Affiliations:** 1 Experimental Neurology, Dept. of Neurology, Goethe University Medical School, Theodor Stern Kai 7, 60590 Frankfurt am Main, Germany; 2 Neurodegeneration Research Laboratory (NDAL), Molecular Biology and Genetics Department, Boğaziçi University, 34470 Istanbul, Turkey; University of Florida, UNITED STATES

## Abstract

The involvement of the ubiquitin-proteasome system (UPS) in the course of various age-associated neurodegenerative diseases is well established. The single RING finger type E3 ubiquitin-protein ligase PARK2 is mutated in a Parkinson’s disease (PD) variant and was found to interact with ATXN2, a protein where polyglutamine expansions cause Spinocerebellar ataxia type 2 (SCA2) or increase the risk for Levodopa-responsive PD and for the motor neuron disease Amyotrophic lateral sclerosis (ALS). We previously reported evidence for a transcriptional induction of the multi-subunit RING finger Skp1/Cul/F-box (SCF) type E3 ubiquitin-protein ligase complex component FBXW8 in global microarray profiling of ATXN2-expansion mouse cerebellum and demonstrated its role for ATXN2 degradation *in vitro*. Now, we documented co-localization *in vitro* and co-immunoprecipitations both *in vitro* and *in vivo*, which indicate associations of FBXW8 with ATXN2 and PARK2. Both FBXW8 and PARK2 proteins are driven into insolubility by expanded ATXN2. Whereas the *FBXW8* transcript upregulation by ATXN2- expansion was confirmed also in qPCR of skin fibroblasts and blood samples of SCA2 patients, a *FBXW8* expression dysregulation was not observed in ATXN2-deficient mice, nor was a *PARK2* transcript dysregulation observed in any samples. Jointly, all available data suggest that the degradation of wildtype and mutant ATXN2 is dependent on FBXW8, and that ATXN2 accumulation selectively modulates *FBXW8* levels, while PARK2 might act indirectly through FBXW8. The effects of ATXN2-expansions on FBXW8 expression in peripheral tissues like blood may become useful for clinical diagnostics.

## Introduction

Spinocerebellar ataxia type 2 (SCA2) is an autosomal dominantly inherited multisystem neurodegenerative disease affecting preferentially the cerebellar Purkinje and brainstem olivo-pontine neurons, the spinal and cortical motor neurons, and the midbrain dopaminergic neurons [1–12]. It is caused by mutations in the gene *ATXN2* localized on chromosome 12q23–24.1 [13] which encodes the protein Ataxin-2 [14–16]. The pathogenic mutations are expansions of an unstable trinucleotide repeat, which normally encodes a domain of 22 glutamines. Thus, SCA2 belongs to a group of polyglutamine (polyQ) disorders including several other spinocerebellar ataxias (SCA1, SCA3, SCA6, SCA7, and SCA17), Huntington’s disease (HD), Dentatorubral-pallidoluysian atrophy (DRPLA), and Spinal and bulbar muscle atrophy (SBMA) [17, 18]. For SCA2 the most common (90% of the human population) polyQ size is 22–23 [15]. Individuals with an *ATXN2* triplet repeat of more than 32 units consisting of pure CAGs probably develop SCA2, with higher repeat sizes leading to earlier manifestation and more severe course of disease [19]. Intermediate repeat sizes between 27 and 31 units that contain interspersed CAA interruptions in *ATXN2* are associated with the risk for other neurodegenerative diseases like Amyotrophic lateral sclerosis (ALS), Frontotemporal dementia (FTD), and Levodopa-responsive idiopathic Parkinson’s disease (PD) [5, 20–24].

Histologically, the polyQ diseases are characterized by accumulation and aggregation of the expanded protein, leading to the formation of insoluble inclusion bodies [25–28]. In SCA2, the ATXN2 protein re-localizes from its normal distribution along the rough endoplasmic reticulum (rER) [29] to accumulate in the cytoplasm [30], while the disease protein in other polyQ disorders (e.g. SCA1, SCA3) usually forms intranuclear inclusions (NIIs) [28, 31]. In an *Atxn2*-CAG42-knockin (KIN) mouse model of SCA2, this cytoplasmic aggregation process was shown to sequester the ATXN2-associated protein PABPC1 into insolubility, selectively in cerebellum at old age [32]. It is thought that accumulation and aggregation may confer toxic gain-of-function properties to the affected protein, thus leading to the selective neurodegeneration patterns with loss of neuronal connectivity and subsequent motor deficits [33, 34]. However, in some of the polyQ diseases the pathogenesis involves also partial loss-of-function effects [35, 36].

The physiological function of ATXN2 is not yet clear, in spite of its selective expression pattern throughout the organism. ATXN2 and its orthologous down to yeast Pbp1 were shown to be involved in the regulation of RNA translation by binding to the poly(A)-binding protein PABPC1 [37, 38]. ATXN2 orthologous have also been implicated in endocytosis [39], stress response [40], cytoskeletal organization [41], neuronal habituation [42, 43], and circadian rhythm [44, 45]. Unfortunately, this knowledge is not sufficient to understand how exactly ATXN2 causes neurodegeneration and the detailed mechanisms remain to be unraveled in order to develop progression biomarkers as well as neuroprotective therapies.

For a further study of disease models and of cellular compensation mechanism, which might be exploited further in the development of therapeutic approaches, we generated the above mentioned *Atxn2*-CAG42-KIN mouse as SCA2 model. In oligonucleotide microarray global transcriptome surveys and in quantitative reverse-transcriptase PCR validation experiments we observed a subsequent +2.5-fold expression upregulation of the E3 ubiquitin-protein ligase component FBXW8, as an effort to avoid the accumulation of ATXN2 protein [32]. The FBXW8 protein is part of a multi-subunit RING-finger type E3 ubiquitin-protein ligase, the so called SCF complex that consists of the RING-finger domain RBX1, the adaptor SKP1, the cullin proteins CUL1 or CUL7, plus one of the F-box-proteins [46]. This complex ensures selective binding of the target protein via the F-box component and associates with the E2 ubiquitin conjugating enzyme via Cullin and RBX1 [47]. The ubiquitinated proteins are then sorted away from their physiological locations and trafficked to the diverse protein degradation pathways of the cell [48]. We formerly showed that expanded ATXN2 is degraded by FBXW8 in an overexpression *in vitro* model of HeLa cells [32].

Now we extended these analyses to a formal demonstration of the FBXW8-ATXN2 association (I) by co-localization studies in HeLa cells, (II) by co-immunoprecipitation studies for both directions of the recombinant tagged proteins in HeLa cells and of the endogenous proteins in mouse cerebellum, (III) by fractionation studies of mouse cerebellum according to protein solubility. Since another single RING-type E3 ubiquitin-protein E3 ligase, PARK2, was previously implicated in ATXN2 degradation, the relative contributions of both factors remained to be clarified. In a study of recombinant tagged proteins transfected in human HEK293 cells, PARK2 had been shown to co-localize with ATXN2, to influence ATXN2 ubiquitylation and to inhibit ATXN2-triggered cell death. In addition, the co-localization of endogenous ATXN2 with PARK2 throughout the cytoplasm of cerebellar Purkinje neurons had been shown, and the tetracyclin-dependent induction of PARK2 in rat PC12 cells was demonstrated to modulate the turnover of ATXN2 [49]. PARK2 is a disease protein responsible for an autosomal recessive juvenile PD variant [50, 51] and a surprisingly large number of putative ubiquitylation substrates of PARK2 has been published on the basis of *in vitro* evidence [52]. In view of a recent publication that PARK2 is responsible for the degradation of the E3 ligase complex component FBXW7 [53], we hypothesized that PARK2 might have broader influences on other E3 ligases and act via FBXW8 turnover to influence ATXN2 degradation. Therefore, we focused on *in vivo* evidence from mouse brain and patient tissues, assessing whether (IV) FBXW8 also interacts with PARK2 in co-immunoprecipitation assays, (V) FBXW8 and/or PARK2 are sequestered into insolubility by expanded ATXN2, (VI) FBXW8 versus PARK2 expression differs in response to ATXN2 mutations. Our results strengthen the importance of FBXW8 in ATXN2 degradation.

## Material and Methods

### Cell culture and transfection

HeLa cells (obtained from DSMZ) were grown in Minimum Essential Medium with Earle’s salt (DMEM, Life Technologies) supplemented with 10% Fetal Bovine Serum Gold (PAA Laboratories), 10 mM Hepes (Sigma) and 1% NEAA (Life Technologies) under standard conditions. Primary skin fibroblasts from SCA2 patients and age- and sex- matched healthy control individuals (CTL) described previously [54] were cultured in Dulbecco’s Modified Eagle Medium (DMEM) containing 4.5 g/l glucose, 2 mM L-glutamine, 100 U/ml penicillin G (all Life Technologies), 100 μg/ml streptomycin (Life Technologies) and 10% Fetal Bovine Serum Gold (PAA Laboratories).

HeLa cells were transfected with one or two of the plasmids stated below. Therefore, 200,000 cells per 6-well or 1.5 million cells per 10-cm dish were seeded 16 h prior to transfection. Transfection was conducted with Effectene Transfection Reagent Kit (Qiagen) using 1 μg (for 6-well) or 5 μg (for 10-cm dish) plasmid DNA according to the manufacturer’s instructions. The transfected cells were incubated for 48 h.

### Co-localization in HeLa cells

HeLa cells were seeded on cover slips in 6-well plates and transfected as described above with the appropriate combinations of the plasmids pCMV-GFP, ATXN2(CAG22)-GFP, ATXN2(CAG74)-GFP, pReceiver-M56 (with Cherry-tag, purchased from Genecopeia) and FBXW8-pReceiver-M55 (with Cherry-tag, Genecopeia). After 48 h, medium was removed and cells were fixed with 70% Ethanol for 20 min. Fixed cells were then washed 5x 1 min with PBS, incubated with 0.25 μg Hoechst per ml PBS for 10 min and again washed 5x 1 min with PBS. Afterwards, the coverslips were mounted onto an object plate with Mountant, PermaFluor (Thermo Scientific). Protein localization was determined by a Zeiss Axiovert 200M inverted microscope equipped with an AxioCam and a 63x oil-immersion objective as well as the Zeiss software AxioVision Rel. 4.8. Images were merged with Image J software.

### Co-immunoprecipitation

For co-immunoprecipitation (Co-IP) studies, HeLa cells were transfected with the corresponding combination of the following plasmids: pCMV-Myc, ATXN2(CAG22)-Myc, ATXN2(CAG74)-Myc [39], eGFP-pReceiver-M07 (with HA-tag, Genecopeia), FBXW8-pEZ-M07 (with 3x HA-tag, Genecopeia) and GFP-Cherry-PARK2 [55]. Two days after transfection, cells were washed once with Dulbecco’s Phosphate Buffered Saline (DPBS, Life technologies) and collected with a cell scraper in 1 ml of DPBS. The resulting cell suspension was centrifuged for 5 min at 5,900 xg, the supernatant was discarded, and cells were lysed in 300 μl of Co-IP buffer [120 mM NaCl; 0.1% Triton X 100; 50 mM Tris‐HCl pH 7.5; Complete Protease Inhibitor Cocktail (Roche)], incubating for 20 min on ice. After centrifugation for 20 min at 15,700 x g, the remaining pellet was discarded and the supernatant transferred to a new tube. For Co-IP studies with cerebellar tissue, 25 mg of tissue were homogenized in 250 μl of NP40 buffer [20 mM Tris‐HCl pH 8.0; 137 mM NaCl; 1% Glycerol; 0.1% Igepal CA-630 (Sigma); 2 mM EDTA; Complete Protease Inhibitor Cocktail (Roche)] with a motor pestle and incubated on ice for 15 min. After centrifugation for 20 min at 16,000 x g and 4°C, the supernatant was transferred to a new tube. For both, cell and tissue lysates, protein concentration was determined with the BCA protein assay kit (Interchim, France). Twenty μl of Protein A-agarose beads (Santa Cruz) were washed twice with lysis buffer [120 mM NaCl; 0.1% Triton X 100; 50 mM Tris‐HCl pH 7.5] for 5 min following centrifugation for 1 min at 2,300 x g. The supernatant was discarded and the beads were incubated for 1 h with 1 ml of blocking buffer [0.2% NaCl; 0.1% Gelatine; 0.05% NaN_3_; 50 mM Tris; 0.1% Triton] at RT on a rotating wheel to decrease the adhesiveness of the beads. At the same time, 200–300 μg of protein extract (HeLa lysate or cerebellar lysate) were pre-incubated with the respective pulling antibodies against ATXN2 (50 μl / sample, self-made) or FBXW8 (5 μl / sample, Sigma) for 1 h at 4°C on a rotating wheel to avoid unspecific binding of the protein to the beads. Subsequently, beads were centrifuged for 1 min at 2,300 x g, the blocking buffer was removed and the protein extract containing the antibody was added. After incubation at 4°C on a rotating wheel overnight, the samples were centrifuged for 1 min at 2,300 x g and the supernatant was removed. To eliminate unbound protein, the samples were washed three times with 1 ml of lysis buffer and centrifuged again for 1 min at 2,300 x g. Afterwards, the supernatant was discarded and 20 μl H_2_O as well as 25 μl of loading buffer were added and the samples were boiled for 5 min at 95°C.

### Probands

A Turkish SCA2 pedigree was studied to identify peripheral tissue effects. Three SCA2 patients (two males and one female, ages 19 to 66 years, ATXN2 CAG repeat expansion sizes 39, 40, 44) were compared with five non-SCA2 first degree relatives (two males and three females, ages 27 to 73 years). SCA2 genotyping was performed as previously reported [56]. Tissue samples were taken after written informed consent and with approvals (E9/06 and 90/08) of the ethics committee of the University Hospital Frankfurt.

### Animals

The generation and characterization of *Atxn2*-CAG42-KIN mice was already described [32]. Expansion of the murine exon 1 of *Atxn2* resulted in an enlarged CAG42 repeat instead of one CAG in wildtype (WT) mice. Mice were backcrossed from mixed 129Sv/Pas×C57BL/6 into C57BL/6 strain for more than 8 generations. Animals were housed under routine health monitoring in individually ventilated cages, fed *ad libitum*, and bred in heterozygous matings. They were sacrificed by cervical dislocation and cerebella were removed in minimal time, frozen immediately in liquid nitrogen, and stored at −80°C. All procedures were done in accordance with the German Animal Welfare Act, the Council Directive of 24 November 1986 (86/609/EWG) with Annex II and the ETS123 (European Convention for the Protection of Vertebrate Animals) at the FELASA-certified Central Animal Facility (ZFE) of the Goethe University Medical School, Frankfurt am Main. Animal studies were approved by the IACUC (steering committee) of the ZFE University Hospital Frankfurt, waiver.

### Genotyping

For genotyping, tail biopsies were taken at ten days of age and DNA was isolated by incubation with Proteinase K (Ambion) and subsequent ethanol precipitation. PCR was performed using 50 ng of DNA, 16.25 μl H_2_O, 2.5 μl 10x Buffer, 4 μl dNTPs (both Takara Bio Inc., Japan), 0.5 μl of forward (5’-*TGA GTT GAC TCC ACA GGG AGG TGA GC*-3’) and 0.5 μl of reverse (5’-CCA TCT CGC CAG CCC GTA AGA TTC-3’) primers and 0.25 μl of LA Taq polymerase (Takara Bio Inc., Japan). The following conditions were applied: 3 min initial denaturation at 94°C, followed by 30 cycles 94°C for 15 s, 68°C for 4 min annealing and elongation, as well as a final elongation step of 9 min at 68°C. The predicted length for the WT and KIN allele is 793 bp and 984 bp, respectively.

### RNA isolation and expression analysis

For expression analysis, RNA was extracted from cerebellar tissue (25 mg) of 18-month-old *Atxn2*-CAG42-KIN or 6-month-old *Atxn2*-knock-out (KO) versus age- and sex-matched WT mice with TRIzol reagent (Invitrogen) and from cells with QIAshredder and RNeasy Mini Kit (both Qiagen) according to the manufacturers’ protocol. For the *in vivo* analysis of SCA2 patients, whole venous blood samples were taken into PAXgene tubes (PreAnalytiX, Switzerland) according to manufacturer’s instructions, stored at −80°C, and RNA-extracted using the PAXgene Blood RNA Kit. Concentration was measured with NanoDrop 2000c UV-Vis Spectrophotometer (Thermo Scientific, USA). Before cDNA synthesis, remaining DNA was digested with DNase I Amplification Grade (Invitrogen). Subsequently, reverse transcription was performed with SuperScript III Reverse Transcriptase (Invitrogen). Expression levels were measured by quantitative real-time reverse-transcriptase PCR (qPCR) with the StepOnePlus Real-Time PCR System (Applied Biosystems) using 25 ng of RNA, 10 μl of FastStart Universal Probe Master (Rox) Mix (Roche) and 1 μl of one of the following TaqMan Assays (Applied Biosystems) for each reaction: *Atxn2* (Mm01199894_m1), *Cul1* (Mm00516318_m1), *Cul7* (Mm00481653_m1), *FBXW8* (Hs00395480_m1), *Park2* (Mm00450186_m1), *Rbx1* (Mm01705487_s1), *Skp1a* (Mm00495559_m1) and *Tbp* (Mm00446973_m1; Hs99999910_m1) as endogenous controls. The PCR conditions were 50°C for 2 min, followed by 10 min at 95°C and 40 cycles of 95°C for 15 s and 60°C for 60 s. Analysis of the gene expression data was conducted using the 2^−ΔΔCt^ method [57]. The Affymetrix oligonucleotide microarray survey of global transcriptomes in cerebella of *Atxn2*-KO mice was deposited in the public database GEO (internet-access at http://www.ncbi.nlm.nih.gov/geo/query/acc.cgi?acc=GSE55177) and is the subject of two independent manuscripts.

### Protein extraction and quantitative immunoblotting

Protein for SDS-polyacrylamide gel electrophoresis (PAGE), immunoblotting, and quantitative densitometry was extracted from 25 mg cerebellar tissue of 18-month-old *Atxn2*-CAG42-KIN and WT mice. For this purpose, tissue was homogenized with a motor pestle in 10 vol. RIPA buffer [50 mM Tris-HCl (pH 8.0); 150 mM NaCl; 1 mM EDTA; 1 mM EGTA; 1% Igepal CA-630 (Sigma); 0.5% sodium deoxycholate; 0.1% SDS; 1 mM PMSF; Complete Protease Inhibitor Cocktail (Roche)] and incubated on ice for 15 min. Primary skin fibroblasts were lysed with the same buffer and treated similar to tissue lysates. After incubation and centrifugation at 4°C and 16.000 xg for 20 min the supernatant was separated and kept on ice until further processing (RIPA fraction). The remaining pellet was dissolved in ½ vol. 2x SDS buffer [137 mM Tris-HCl (pH 6.8); 4% SDS; 20% glycerol; Complete Protease Inhibitor Cocktail (Roche)] by sonification. After 10 min of centrifugation at 16.000 xg the pellet was removed and the supernatant represented the SDS fraction. Protein concentrations of both fractions were measured with the BCA protein assay kit (Interchim, France) and normalized to the respective buffers. For SDS-PAGE, the samples were boiled with 2x Loading Buffer [25% stacking gel buffer (0.5M Tris, 0.4% SDS; pH 6.8), 20% Glycerol, 4% SDS, 5% β-Mercaptoethanol and 0.05% Bromophenol blue] at 95°C for 5 min and 20 μg of each sample were loaded onto a 7.5% polyacrylamide gel. After gel electrophoresis, proteins were transferred to a PVDF membrane. Membranes were blocked with 5% slim milk powder in PBST and incubated with primary antibodies against ATXN2 (1:400, BD Transduction Laboratories, 611378, monoclonal, host: mouse), FBXW8 (1:600, Pierce Antibodies, **HPA038851,** polyclonal, host: rabbit), PARK2 (1:800, Cell Signaling, 2132S, polyclonal, host: rabbit), or β-ACTIN (1:10,000, Sigma, A5441, monoclonal, host: mouse). Proteins were detected with SuperSignal West Pico (Thermo Scientific), with varying exposure times to avoid film sensitivity or saturation problems as well as non-linear effects. The images were digitalized on a scanner (Epson) and densitometry was performed with ImageJ software. After normalization of candidate protein values versus β-ACTIN values from the identical membrane in EXCEL, the changes were evaluated in GraphPad statistics and plotting.

### Immunohistochemical staining

Paraffin-embedded slices were then rehydrated by incubation in a descending ethanol series and stored in Tris/HCl buffer pH 7.6. Slices were set into a container filled with Bull’s Eye Decloaker (1:20) in the autoclave (Biocare Medical). Conditions were as follows: 125°C for 30 s and 90°C for 10 s. Slides were subsequently cooled down and washed in Tris/HCl pH7.6. For background reduction, slices were incubated with 100% methanol, 30% H_2_O_2_ and Tris/HCl pH 7.6 in the proportion 1:1:8 for 30 min in a wet chamber. Afterwards, they were washed in Tris/HCl pH 7.6 and blocked in 2.5 μl Triton-X-100, 18.2 mg DL-Lysine and 998 μl 5% Tris-BSA for 30 min. Incubation with the first antibodies (anti-ATXN2, BD Biosciences, 611378, 1:50 and anti-FBXW8, Prestige, HPA038851, 1:20) took place overnight. After another washing step, slices were incubated with the secondary fluorescently-labeled antibodies (Cy3 and Cy2, Dianova, 711-225-152 and 715-165-150, 1:1000) for six hours and mounted with DAKO fluorescent mounting medium. Microscopic pictures were taken with a Nikon confocal microscope Eclipse 90i and a 60x magnification.

### Statistical analysis

Data were analyzed with GraphPad Prism software version 4.03 (2005) using Student’s *t* test. Error bars indicate SEM. Significant *p* values (<0.05) were marked as follows: *p*<0.05 *, *p*<0.01 **, *p*<0.001 ***. A trend (T) was noted when 0.05<*p*<0.1.

## Results

### FBXW8 co-localizes and interacts with normal and expanded ATXN2 in HeLa cells

**Fig 1:**
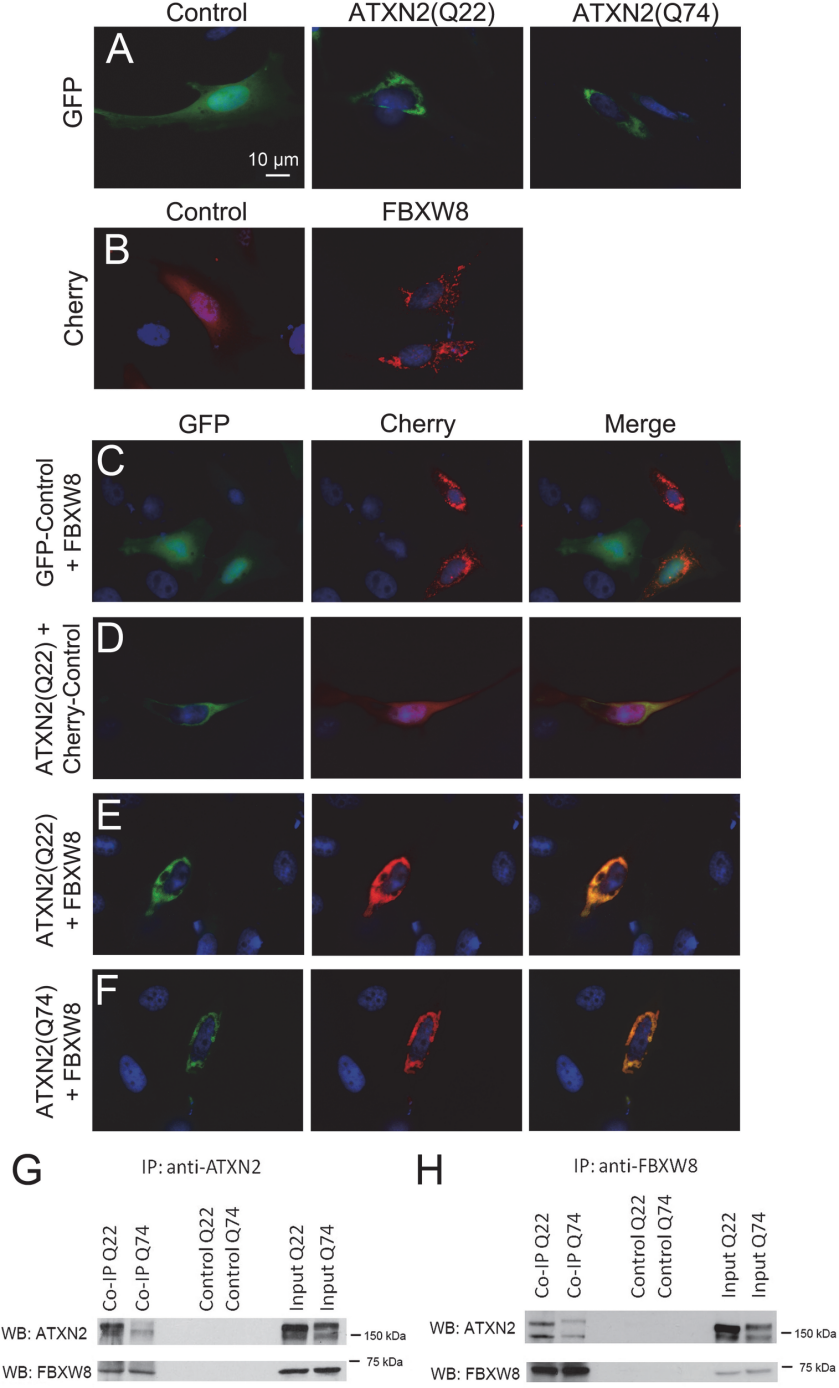
FBXW8 and ATXN2 co-localize when they are transiently overexpressed in HeLa cells. Single transfections of GFP-control, ATXN2(Q22)-GFP and ATXN2(Q74)-GFP (A) as well as Cherry-control and FBXW-Cherry (B) show cytoplasmatic expression of the fluorescent proteins compared to a distinct signal of ATXN2 and FBXW8 plasmids. Double transfections of GFP-control and FBXW8-Cherry do not show co-localizing fluorescent signals (C), similar to the double transfection of ATXN2(Q22)-GFP and Cherry-control (D). ATXN2(Q22)-GFP (E) and ATXN2(Q74)-GFP (F) co-localize with FBXW8-Cherry in double transfections. Both proteins are located in the cytoplasm (one experiment, n = 4–10 cells per transfection). In transfected HeLa cells pulling with either anti-ATXN2 (G) or anti-FBXW8 antibody (H) results in detection of both proteins in the Co-IP lysates, indicating their interaction. The interaction is independent of the polyQ length (experiment repeated once for anti-ATXN2 and twice for anti-FBXW8, representative images).

In order to assess if ATXN2 and FBXW8 interact and in which subcellular compartment they meet, we performed Co-IP as well as co-localisation studies using human HeLa cells. ATXN2 was previously reported to localize along the rER, after overexpression to the trans-Golgi network, to shuttle into the nucleus, to act at the plasma membrane, and to redistribute to stress granules during periods of starvation or oxidative stress [29, 39, 40, 58, 59]. For the co-localisation, FBXW8-Cherry (red fluorescence) and either ATXN2(Q22)-GFP or ATXN2(Q74)-GFP (green fluorescence) were transiently co-transfected, and their distribution overlap was studied using fluorescence microscopy. To avoid unspecific staining, control plasmids containing either a Cherry- or a GFP-tag were used. Fig. 1A and B show the non-targeted, cytoplasmatic expression of both fluorescent proteins (left panel) compared to the specific signal resulting from the transfections with ATXN2(Q22)-GFP and AXN2(Q74)-GFP as well as FBXW8-Cherry (middle and right panels). Aggregates of ATXN2 were not observed in these cells. HeLa cells are in contrast to neuronal cells less prone to aggregation and in this approach the overexpression level was too low and incubation time too short for the formation of distinct aggregates. The localisation of ATXN2 around the nucleus is clearly distinctive from the scattered distribution of FBXW8 in these single transfections. Fig. 1C and D show the result of the double transfection of FBXW8-Cherry together with the GFP-control plasmid as well as the double transfection of ATXN2(Q22)-GFP together with the Cherry-control plasmid. These images prove on the one hand that the localisation of FBXW8 is not changed due to any side effect of the GFP-plasmid. On the other hand, they reveal that although the background signal of the Cherry-control plasmid is almost everywhere in the cell it does not show selective co-localization with the GFP-signal of ATXN2(Q22). This is in contrast to the almost complete co-localization with cytoplasmic selectivity in the double transfections of ATXN2(Q22)-GFP or ATXN2(Q74)-GFP together with FBXW8-Cherry as can be seen in Fig. 1E and F, respectively. Thus, ATXN2 co-localizes with FBXW8 in the cytoplasm of HeLa cells without detectable difference between normal and expanded ATXN2.

To confirm that ATXN2 and FBXW8 not only co-localize but also associate in a protein complex, we conducted co-immunoprecipitation studies using overexpressed ATXN2(Q22)-myc or ATXN2(Q74)-myc and FBXW8-HA in HeLa cells. Pulling with an anti-ATXN2 antibody we could detect two bands for ATXN2 in the input as well as in the Co-IP (Fig. 1G and S1 Fig.). The smaller band (∼150 kDa) represents the endogenous protein (alternative splicing with skipping of exons is known to occur and two possible translation start codons exist) [60, 61], the larger band represents the overexpressed recombinant maximal-length ATXN2 band (including the myc tag), with a size shift visible for the larger polyQ expansion. In the Co-IP Q74 lane, the endogenous wild-type ATXN2 of HeLa cells was clearly visible, while the upper band of overexpressed mutant ATXN2 was only faint, but this may be due to the overexpression of Q74 being weaker than of Q22 in the input, and to decreased solubility of precipitated Q74 ATXN2. No bands could be detected in the negative control, neither for ATXN2 nor for FBXW8. In the input as well as in the Co-IP, a band around 75 kDa was detected for FBXW8 showing that ATXN2 and FBXW8 indeed interact with each other, independent of the length of the polyQ repeat. When pulling with FBXW8, again one band for FBXW8 as well as two bands for ATXN2 could be detected in the input and in the Co-IP while there was no band in the negative control (Fig. 1H and S1 Fig.). ATXN2(Q74) bands in the Co-IP as well as in the input were weaker than ATXN2(Q22), while endogenous wild-type ATXN2 (lower band) co-precipitated more readily. ATXN2(Q74) bands in the Co-IP as well as in the were weaker compared to ATXN2(Q22), but already in the input wildtype ATXN2 displayed a stronger signal. Thus, human recombinant ATXN2 and FBXW8 showed a consistent protein association, demonstrable in both directions for transiently transfected HeLa cells, without dependence on the polyQ expansion.

### FBXW8 interacts with ATXN2 and accumulates in the insoluble fraction of *Atxn2*-CAG42-KIN cerebellum

We then assessed whether this interaction can be detected also for the endogenous proteins in cerebellar brain tissue. In cerebellar Purkinje neurons, ATXN2 exhibited the known diffuse cytoplasmic distribution, while FBXW8 was detected in granular foci of the cytoplasm, both for WT and mutant mice. A potentially increased recruitment of expanded ATXN2 into these foci could not be demonstrated even in 24-month-old mice (S2 Fig.). Furthermore, ubiquitinated inclusion bodies in mutant Purkinje neurons were also not detectable (data not shown). This is probably due to the relatively small CAG expansion and the mild phenotype within the lifespan of these *Atxn2*-CAG42-KIN mice, where cytoplasmic inclusion bodies of ATXN2 remain hard to visualize in Purkinje cells until old age.

**Fig 2:**
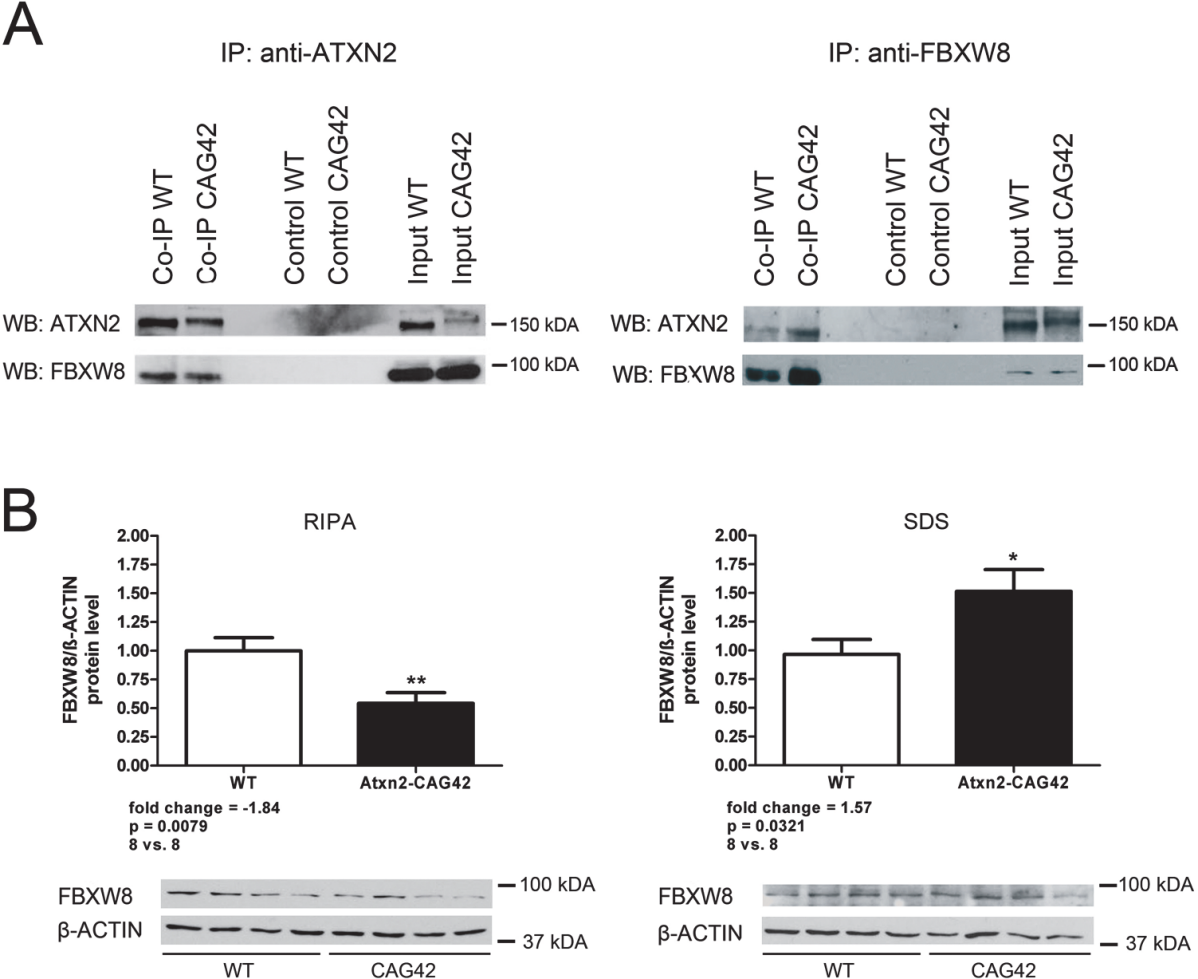
FBXW8 is shifted into insolubility in *Atxn2*-CAG42-KIN mice due to interaction with expanded ATXN2. (A) Pulling either with anti-ATXN2 or anti-FBXW8 antibody, ATNX2 and FBXW8 show an interaction in the cerebellum of 18-month-old *Atxn2*-CAG42-KIN mice independent of the polyQ length (experiment repeated three times for anti-ATXN2 and once with anti-FBXW8, representative images). (B) In cerebellar tissue of 18-month-old *Atxn2*-CAG42-KIN mice FBXW8 protein level is downregulated in the RIPA-soluble fraction while it is upregulated in the SDS-soluble fraction (two independent experiments each with 4 *Atxn2* ^CAG1/CAG1^ vs. 4 *Atxn2* ^CAG42/CAG42^ mice).

Next, we performed co-immunoprecipitation assays with WT and *Atxn2*-CAG42-KIN mouse cerebellar protein lysates pulling either with an anti-ATXN2 or an anti-FBXW8 antibody (Fig. 2A and S3 Fig.). In both cases, ATXN2 bands can be seen in the input as well as in the Co-IP lanes with the KIN band slightly above the WT band due to the CAG expansion. FBXW8 is also detectable in the input and Co-IP lanes in both approaches. No bands for ATXN2 or FBXW8 are visible in the negative controls where beads were incubated without any antibody but lysate only. These data confirm that the interaction of endogenous ATXN2 and FBXW8 in WT and *Atxn2*-CAG42-KIN mouse cerebella occurs independently of the CAG repeat length.

For another ATXN2-interacting protein, PABPC1, we already reported that ATXN2 polyQ expansion drives it into insolubility [32]. Therefore, we investigated whether the solubility of FBXW8 in *Atxn2*-CAG42-KIN mice is altered as well. For this purpose we quantitatively measured FBXW8 protein levels in the tissue fraction readily soluble in RIPA-buffer versus the fraction only soluble in SDS. The FBXW8 protein level in the RIPA fraction of *Atxn2*-CAG42-KINs was significantly decreased compared to WT levels (-1.84-fold, p-value = 0.0079), while it was significantly increased (+1.59-fold, p-value 0.0321) in the SDS fraction (Fig. 2B and S3 Fig.; combination of two independent experiments each with 4 *Atxn2* ^CAG1/CAG1^ vs. 4 *Atxn2* ^CAG42/CAG42^ mice). Taken together, all results suggest that the ATXN2 and FBXW8 meet in the cytoplasm of disease-susceptible cerebellar neurons *in vivo* and together redistribute to relatively insoluble fractions of mutant tissue, in the absence of visible inclusion bodies.

### PARK2 interacts with FBXW8 and becomes insoluble in *Atxn2*-CAG42-KIN cerebellum

**Fig 3:**
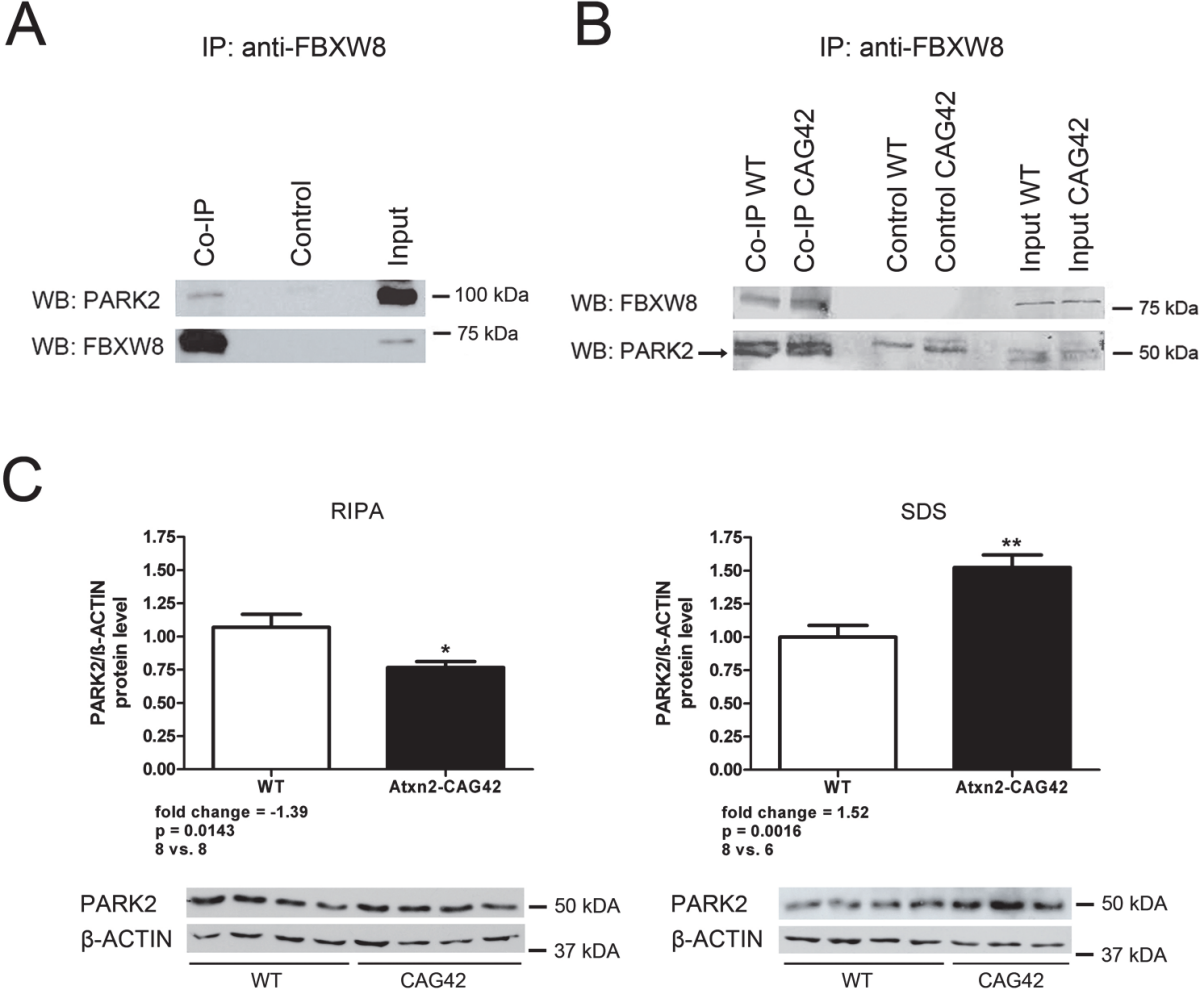
PARK2 interacts with FBXW8 and is recruited into insolubility in *Atxn2*-CAG42-KIN mice. (A) In HeLa cells overexpressing Cherry-GFP-PARK2 and FBXW8-HA, pulling with anti-FBXW8 antibody resulted in the detection of FBXW8 as well as of PARK2 in Co-IP lysates, demonstrating their interaction (experiment repeated twice, representative image). (B) PARK2 interacts with FBXW8 in Co-IP samples of *Atxn2*-CAG42-KIN mice independent of the polyQ length. Lower bands represent PARK2 protein (experiment repeated once). (C) PARK2 protein level is decreased in the RIPA-soluble fraction while it is increased in the SDS-soluble fraction (8 *Atxn2* ^CAG1/CAG1^ mice vs. ≥ 6 *Atxn2* ^CAG42/CAG42^ mice).

A protein association of ATXN2 with the ubiquitin E3 ligase PARK2 (PARKIN) and a modulation of ATXN2 degradation by PARK2 were previously observed [49]. PARK2 is known to be responsible for the degradation of FBXW7, another F-box protein implicated in the degradation of c-Myc and Cyclin-E, regulating growth and cell cycle [46, 53]. Thus, a cascade was proposed previously, where the neuronal target protein is selectively recognized by the F-box protein as part of a SCF ubiquitin E3 ligase complex, while the degradation of the F-box protein itself depends on the higher-order ubiquitin E3 ligase PARK2 [53]. In an alternative scenario, it had been also been claimed that PARK2 could bind to FBXW7 / hSEL-10 as functional part of the SCF complex [46, 62]. To now test the association of PARK2 with FBXW8, we first studied the recombinant proteins in HeLa cells. FBXW8-HA and GFP-Cherry-PARK2 plasmids were used for transient overexpression and the anti-FBXW8 antibody was used for immunoprecipitation (Fig. 3A and S4 Fig.). The double tag of PARK2 was necessary to avoid an overlay of the PARK2 band with the FBXW8 antibody heavy chain, as both antibodies were produced in rabbit. In the immunoblots we could detect FBXW8 at the height of 75 kDa in the input as well as in the Co-IP lane but not in the negative control, demonstrating the specificity of the immunoprecipitation. PARK2 with both tags was detected at ∼100 kDa in the input and the Co-IP but not in the negative control. These HeLa data with overexpression of recombinant tagged proteins suggest that FBXW8 and PARK2 interact.

To further substantiate this interaction *in vivo* for the disease-susceptible tissue, we used cerebella from *Atxn2*-CAG42-KIN mice again. Pulling with the anti-FBXW8 antibody we could confirm the interaction with PARK2 (Fig. 3B and S4 Fig.). A band at ∼75 kDa for FBXW8 in the input and the Co-IP lanes but no band in the negative control demonstrates the immunoprecipitation efficiency. There are two bands at the height of ∼50 kDa where the PARK2 band should be located. Only the smaller band reflects the co-immunoprecipitation of PARK2, while the larger band is also detectable in the negative control indicating an unspecific signal. These data suggest that PARK2 associates with FBXW8 and ATXN2 in one protein complex.

To test whether PARK2 is also sequestered into insolubility together with FBXW8 by the ATXN2 polyQ expansion, quantitative immunoblots were performed to document the PARK2 protein levels in RIPA-soluble and SDS-soluble fractions of *Atxn2*-CAG42-KIN cerebella (combination of two independent experiments with 4 *Atxn2* ^CAG1/CAG1^ mice vs. 3 and 4 *Atxn2* ^CAG42/CAG42^ mice, respectively). The results in Fig. 3C and S4 Fig. show that PARK2 is significantly downregulated (-1.39-fold, p-value 0.0143) in the RIPA fraction while it is significantly upregulated (+1.52-fold, p-value 0.0016) in the SDS fraction. However, *Park2* transcript expression remained unchanged in cerebellar tissue of *Atxn2*-CAG42-KIN mice (S5 Fig.; 10 *Atxn2* ^CAG1/CAG1^ mice vs. 10 *Atxn2* ^CAG42/CAG42^ mice). These results suggest that the previously documented accumulation, insolubility, and aggregation of expanded ATXN2 in aged mouse cerebellum affect FBXW8 and PARK2 similarly, but that the transcriptional expression regulation of *Park2* is less dependent on expansions of the putative substrate ATXN2 than the expression regulation of *Fbxw8*. These findings strengthen the role of PARK2 in SCA2 pathology and demonstrate a new protein interaction partner for FBXW8.

### *Fbxw8* upregulation in mouse cerebellum is a specific effect caused by Atxn2 toxic gain of function

In order to examine the specificity of the *Fbxw8* expression upregulation in *Atxn2*-CAG42-KIN mice, we also analyzed other components of the SCF complex on transcript level. Again, cerebellar tissue of 18-month-old WT and mutant animals was used to measure *Cul1*, *Cul7*, *Rbx1*, *Skp1* and *Park2* levels via RT-qPCR. However, none of the transcripts showed any change (S5 Fig.). Furthermore, oligonucleotide microarray data from cerebellar tissue of 6-month-old *Atxn2*-KO mice with an obesity phenotype [63, 64] documented transcript levels of *Fbxw8* (oligonucleotide spot 1436732_s_at, log2-fold-change 0.16) and *Park2* (oligonucleotide spot 1449975_a_at, log2-fold-change 0.28) without significant alterations. These results suggest that the upregulation of *Fbxw8* in *Atxn2*-CAG42-KIN mice is specific for polyQ-expanded ATXN2. This may be due to the function of FBXW8 as a substrate specific target recognition subunit.

### FBXW8, but not *PARK2* expression changes in SCA2 patient fibroblasts and blood

**Fig 4:**
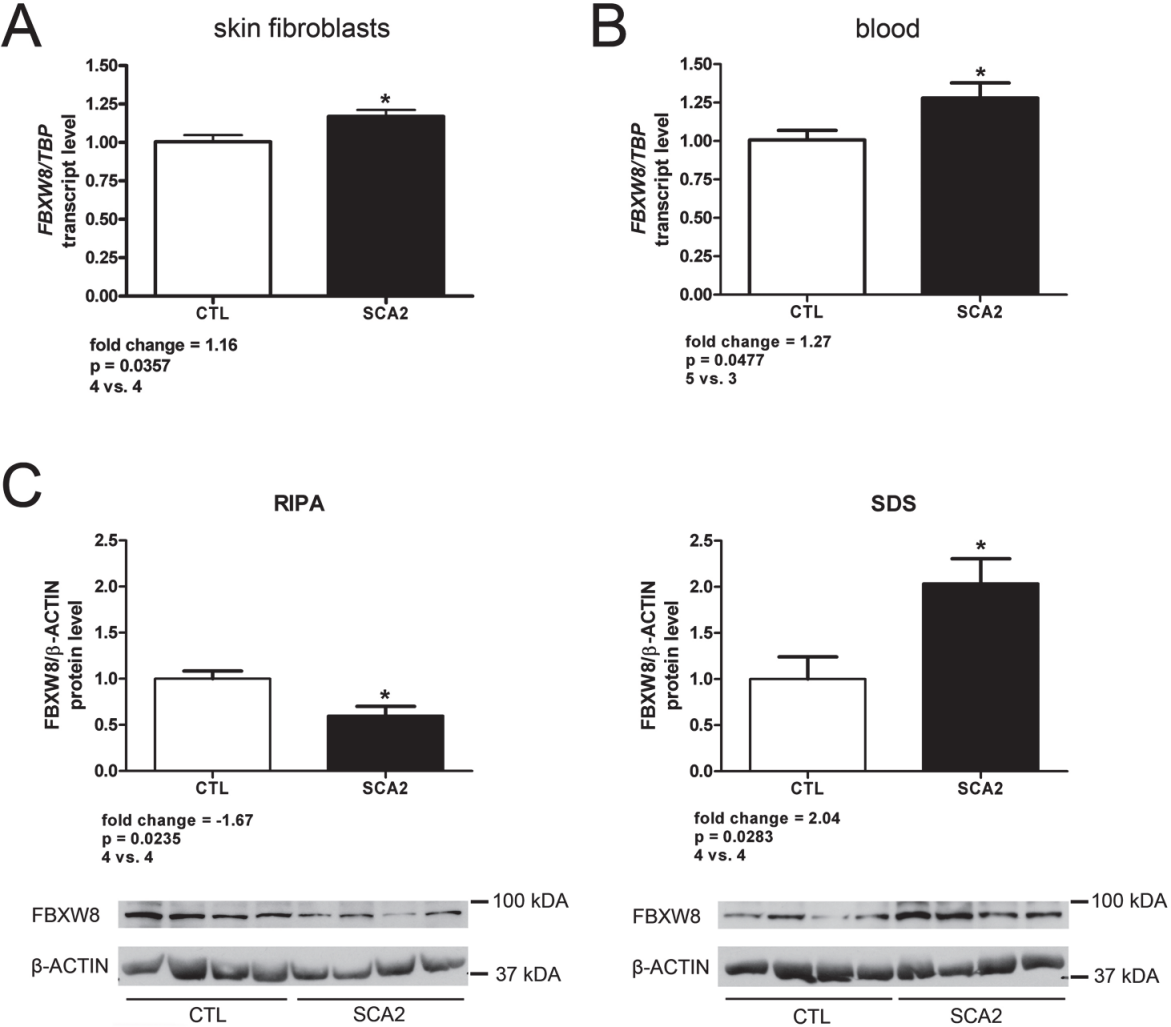
FBXW8 protein levels are dysregulated in SCA2 patient material. FBXW8 expression is upregulated at the transcript level in SCA2 patient skin fibroblasts (A; 4 CTL individuals vs. 4 SCA2 patients) as well as in SCA2 patient blood samples (B; 5 CTL individuals vs. 3 SCA2 patients). (C) At the protein level FBXW8 is decreased in the RIPA-soluble fraction while it is increased in the SDS-soluble fraction in SCA2 patient fibroblasts (4 CTL individuals vs. 4 SCA2 patients).

To investigate in patient material, if FBXW8 plays a role in SCA2 pathogenesis and particularly in peripheral tissues, we employed primary skin fibroblasts as well as whole blood samples. Firstly, transcription levels of *FBXW8* were studied to test if there is a similar upregulation as in the brain of the SCA2 mouse model. Fig. 4A shows the slight but significant upregulation of *FBXW8* in patient skin fibroblasts with a fold change of +1.16 (p-value 0.0357, 4 controls vs. 4 SCA2 patients) observed by RT-qPCR. A similar upregulation was found in blood of SCA2 patients (5 controls vs. 3 SCA2 patients) with a fold change of +1.27 (p-value 0.0477, Fig. 4B). In contrast, significant changes of *Park2* transcript levels were not detected in qPCR analyses of SCA2 patient skin fibroblasts (data not shown) similar to the results observed in *Atxn2*-CAG42-KIN cerebellum. Thus, also in peripheral tissues *FBXW8* rather than *PARK2* mRNA levels are modulated by polyQ expansions of ATXN2 and may thus be relevant for clinical diagnostics.

To further evaluate the distribution of FBXW8, protein extracts from the skin fibroblasts were isolated and studied in quantitative immunoblots. Similar to the previous data in cerebella of our SCA2 mouse model, a significant decrease of FBXW8 in the RIPA-soluble fraction to about half (-1.67-fold, p-value 0.0235) of control levels was observed, while a significantly increased abundance to doubled levels (+2.04-fold, p-value 0.0283) was found in the SDS-soluble fraction (4 controls vs. 4 SCA2 patients) (Fig. 4C and S6 Fig.). On protein level, PARK2 could not be quantified in SCA2 patient fibroblasts due to its low abundance in skin. These results confirm that the sequestration of FXBW8 by expanded ATXN2 into insolubility occurs in SCA2 patient peripheral tissues similarly as in the brain of our SCA2 mouse model.

## Discussion

Eukaryotic cells have primarily two mechanisms to degrade toxic proteins: the ubiquitin-proteasome system (UPS) and autophagy. While autophagy mainly occurs in the cytoplasm and attacks long-lived and aggregated proteins, the UPS degrades short-lived and misfolded proteins in the cytoplasm as well as in the nucleus [65]. The relevance of the UPS for polyQ expansion and other neurodegenerative diseases has been discussed in numerous publications [66–69]. As many mutant proteins are misfolded they should normally be degraded by the UPS, however, within aggregates they become too bulky for the proteasome and then autophago-lysosomal degradation becomes necessary [70]. Nevertheless, the ubiquitination step is always necessary and occurs also at advanced stages of aggregation and old age [71]. Regarding SCA2, we previously found in a mouse model of the initial mild disease stage that the polyQ expanded ATXN2 becomes progressively insoluble in the disease-susceptible cerebellar tissue and sequestrates its protein interactors [32]. Here we showed that this initial disease process sequestrates the two ubiquitin E3 ligase complex components FBXW8 and PARK2, which had already been shown to modulate ATXN2 degradation. A recruitment of cellular proteins like VCP and ubiquitin to aggregates has recently been shown for Ataxin-3, another polyQ protein responsible for SCA3 [72]. The insolubility of FBXW8 can also be observed in peripheral tissue, where PARK2 protein is undetectable, as a pathological event during later disease stages in SCA2. The selective upregulation of *FBXW8* transcript levels is a subtle, but very consistent finding and might become relevant for the search of blood biomarkers for SCA2. The fold-change of this upregulation is smaller in the peripheral tissues than in brain. This can probably be explained since the polyQ expansion of ATXN2 inherently alters the conformation and relative solubility of ATXN2 and of its direct interactor proteins, while the subsequent polyQ aggregation process depends on specific neuronal differentiation and on calcium-mediated excitation, a weak phenomenon during the short lifespan and culture period of peripheral cells [73]. Thus, the accumulation and insolubility of expanded ATXN2 in such cells will not progress much, in contrast to processes in postmitotic neurons throughout a life-time.

It is unclear why two ubiquitin E3 ligases are involved in ATXN2 degradation, and how they distribute tasks. For a definite elucidation of the mechanism, laborious cell-free assays of ubiquitination with purified protein fragments might be crucial, which are clearly beyond the scope of this tissue-focused manuscript. Such purification efforts would be cumbersome in view of the limited expression of both proteins in tumor cell lines and the 140 kDa size of ATXN2, and it might be impossible to model the complex effects of all splicing variants of PARK2 [74] and of the stress-triggered redistribution from the cytoplasm to stress granules in the case of ATXN2 and from the cytoplasm to mitochondria in the case of PARK2. Our tissue observation that the endogenous expression regulation of *Fbxw8*, but not *Park2* or other SCF components responds to ATXN2 polyQ expansions may reflect the dependence of FBXW8 abundance on relatively few substrates. The findings are compatible with the hierarchy of the ubiquitination enzymes, where only 16 subtypes of E1, 53 subtypes of E2, and 527 subtypes of E3 enzymes [69] reflect increasing target-specificity, and where PARK2 may serve as a higher-order E3 ligase enzyme responsible for the ubiquitination and degradation of lower-order E3 ligases such as FBXW7 [53] and FBXW8, which mediate maximal target-specificity.

In conclusion, our data implicate FBXW8 insolubility and transcriptional upregulation as a specific and early cellular response to SCA2 pathogenesis. Consistent observations were made in cerebellum of a SCA2 mouse model and in peripheral tissues of SCA2 patients. This might become relevant for the clinical diagnostics in patients with SCA2, ALS, FTD, and PD. Since FBXW8 is able to reduce the accumulation of expanded ATXN2, it also represents a promising therapeutic target. Further biochemical experiments will have to elucidate the exact functional role of FBXW8 in relation to PARK2, and the involvement of diverse protein domains and crucial amino acids in these protein interactions.

## Supporting Information

S1 Fig FBXW8 and ATXN2 interact when they are transiently overexpressed in HeLa cells. Larger cutout of Western Blot images from Fig. 1. In transfected HeLa cells pulling with either anti-ATXN2 (G) or anti-FBXW8 antibody (H) results in detection of both proteins in the Co-IP lysates, indicating their interaction. The interaction is independent of the polyQ length (experiment repeated once for anti-ATXN2 and twice for anti-FBXW8, representative images).
(TIF)

S2 Fig FBXW8 co-localization with ATXN2 in mouse cerebellum. Double immunofluorescence analysis detected ATXN2 in cerebellar Purkinje neurons throughout the cytoplasm, whereas FBXW8 was detected in cerebellar Purkinje neurons within discrete foci of the cytoplasm, irrespective of the ATXN2 polyQ expansion status.
(TIF)

S3 Fig FBXW8 is shifted into insolubility in *Atxn2*-CAG42-KIN mice due to interaction with expanded ATXN2. Larger cutout of Western Blot images from Fig. 2. (A) Pulling either with anti-ATXN2 or anti-FBXW8 antibody, ATNX2 and FBXW8 show an interaction in the cerebellum of 18-month-old *Atxn2*-CAG42-KIN mice independent of the polyQ length (experiment repeated three times for anti-ATXN2 and once with anti-FBXW8, representative images). (B) In cerebellar tissue of 18-month-old *Atxn2*-CAG42-KIN mice FBXW8 protein level is downregulated in the RIPA-soluble fraction while it is upregulated in the SDS-soluble fraction (two independent experiments each with 4 *Atxn2* ^CAG1/CAG1^ vs. 4 *Atxn2* ^CAG42/CAG42^ mice).
(TIF)

S4 Fig PARK2 interacts with FBXW8 and is recruited into insolubility in *Atxn2*-CAG42-KIN mice. Larger cutout of Western Blot images from Fig. 3. (A) In HeLa cells overexpressing Cherry-GFP-PARK2 and FBXW8-HA, pulling with anti-FBXW8 antibody resulted in the detection of FBXW8 as well as of PARK2 in Co-IP lysates, demonstrating their interaction (experiment repeated twice, representative image). (B) PARK2 interacts with FBXW8 in Co-IP samples of *Atxn2*-CAG42-KIN mice independent of the polyQ length. Lower bands represent PARK2 protein (experiment repeated once). (C) PARK2 protein level is decreased in the RIPA-soluble fraction while it is increased in the SDS-soluble fraction (8 *Atxn2* ^CAG1/CAG1^ mice vs. ≥ 6 *Atxn2* ^CAG42/CAG42^ mice).
(TIF)

S5 Fig Transcript levels of other SCF components remain unchanged in *Atxn2*-CAG42-KIN mice. In contrast to *Fbxw8*, transcript levels of *Cul1*, *Cul7*, *Rbx1*, *Skp1* (A) and *Park2* (B) are not significantly changed in cerebella of 18-month-old *Atxn2*-CAG42-KIN mice (≥ 8 *Atxn2* ^CAG1/CAG1^ mice vs. ≥ 8 *Atxn2* ^CAG42/CAG42^ mice). n.s. = non-significant. T = trend.
(TIF)

S6 Fig FBXW8 protein levels are dysregulated in SCA2 patient material. Larger cutout of Western Blot images from Fig. 4. At the protein level FBXW8 is decreased in the RIPA-soluble fraction while it is increased in the SDS-soluble fraction in SCA2 patient fibroblasts (4 CTL individuals vs. 4 SCA2 patients).
(TIF)
