# Supplementary figures and images for: Both Ubiquitin Ligases FBXW8 and PARK2 Are Sequestrated into Insolubility by ATXN2 PolyQ Expansions, but Only FBXW8 Expression Is Dysregulated
Source: PLoS One. 2015 Mar 19;10(3):e0121089. doi: 10.1371/journal.pone.0121089 (PMC4366354; doi:10.1371/journal.pone.0121089)

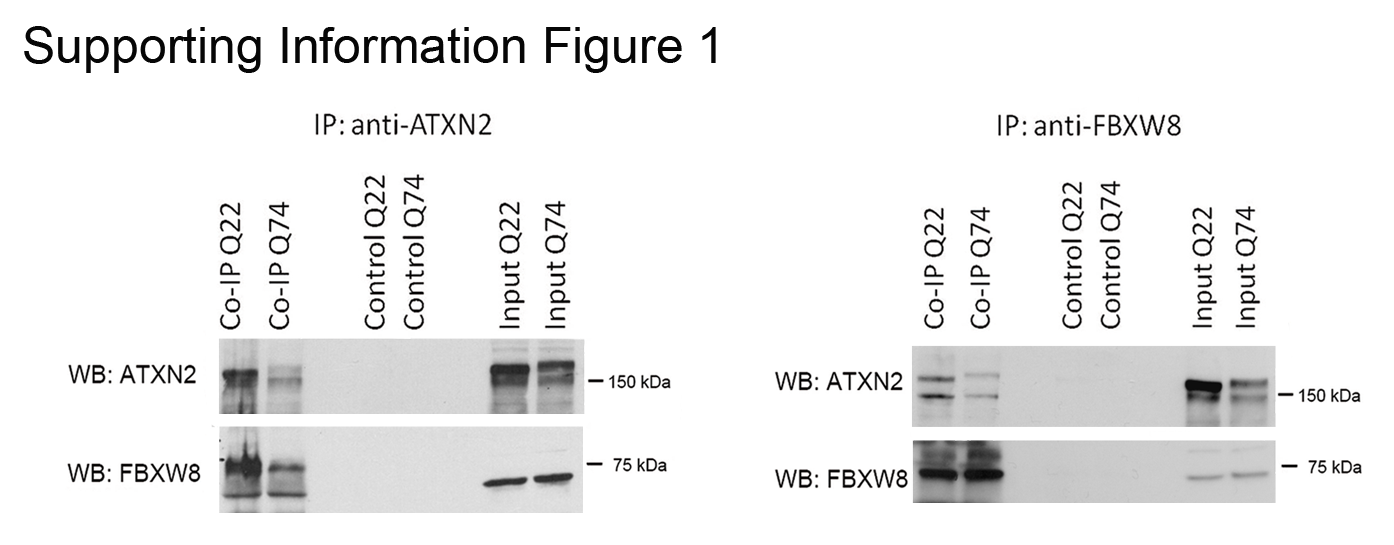

Supplement: S1 Fig — Larger cutout of Western Blot images from Fig. 1. In transfected HeLa cells pulling with either anti-ATXN2 (G) or anti-FBXW8 antibody (H) results in detection of both proteins in the Co-IP lysates, indicating their interaction. The interaction is independent of the polyQ length (experiment repeated once for anti-ATXN2 and twice for anti-FBXW8, representative images). (TIF) [file pone.0121089.s001.tif]

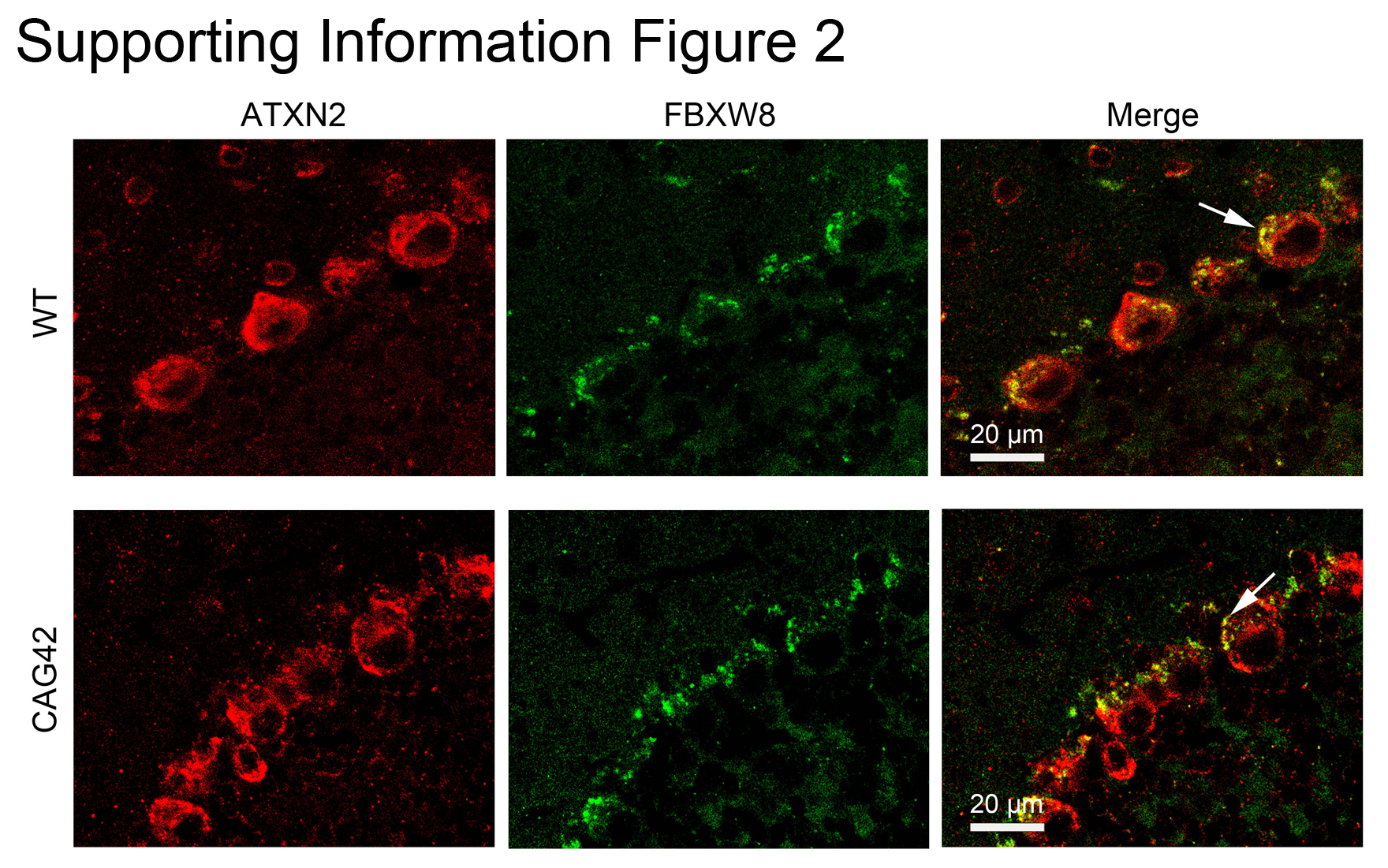

Supplement: S2 Fig — Double immunofluorescence analysis detected ATXN2 in cerebellar Purkinje neurons throughout the cytoplasm, whereas FBXW8 was detected in cerebellar Purkinje neurons within discrete foci of the cytoplasm, irrespective of the ATXN2 polyQ expansion status. (TIF) [file pone.0121089.s002.tif]

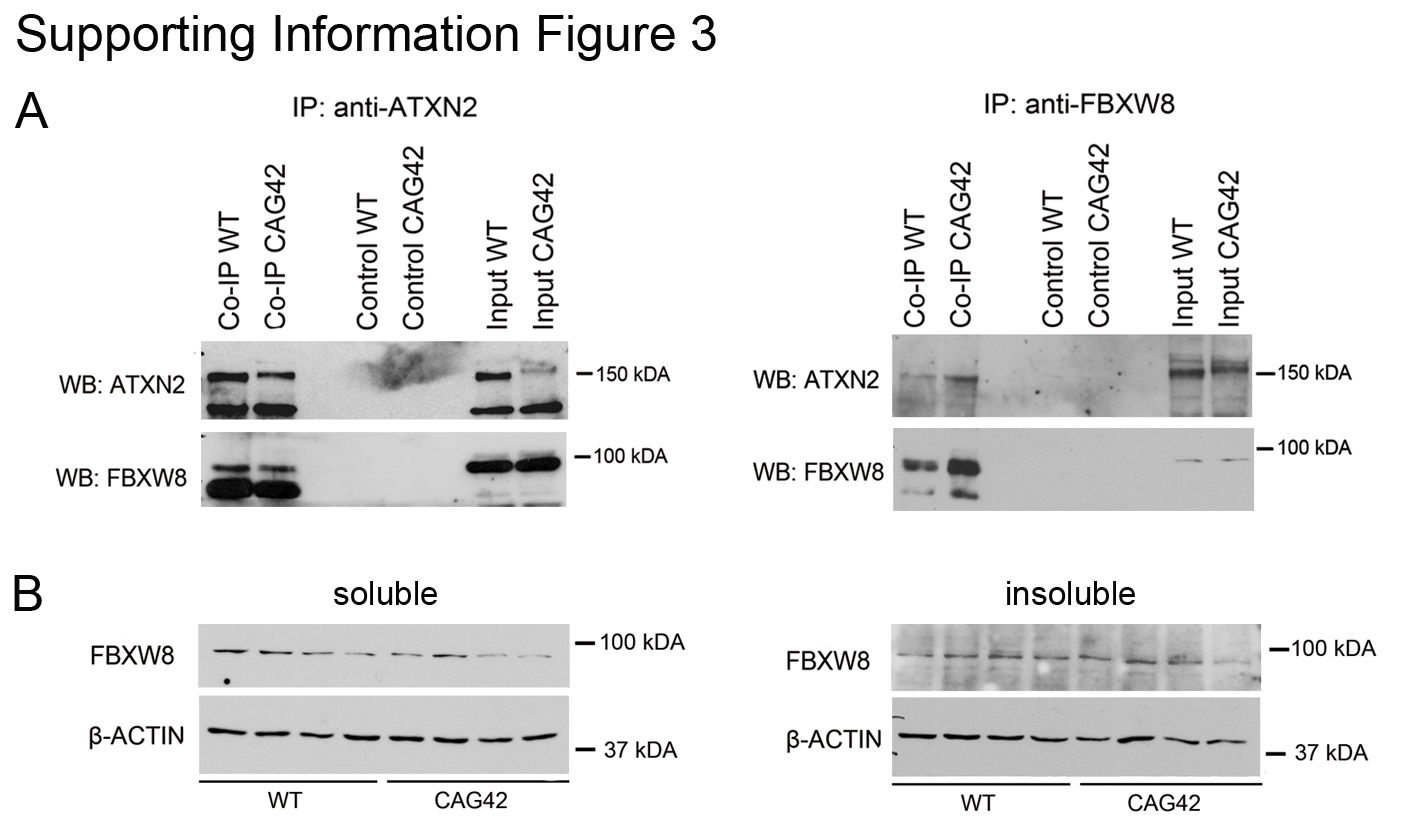

Supplement: S3 Fig — Larger cutout of Western Blot images from Fig. 2. (A) Pulling either with anti-ATXN2 or anti-FBXW8 antibody, ATNX2 and FBXW8 show an interaction in the cerebellum of 18-month-old Atxn2-CAG42-KIN mice independent of the polyQ length (experiment repeated three times for anti-ATXN2 and once with anti-FBXW8, representative images). (B) In cerebellar tissue of 18-month-old Atxn2-CAG42-KIN mice FBXW8 protein level is downregulated in the RIPA-soluble fraction while it is upregulated in the SDS-soluble fraction (two independent experiments each with 4 Atxn2 CAG1/CAG1 vs. 4 Atxn2 CAG42/CAG42 mice). (TIF) [file pone.0121089.s003.tif]

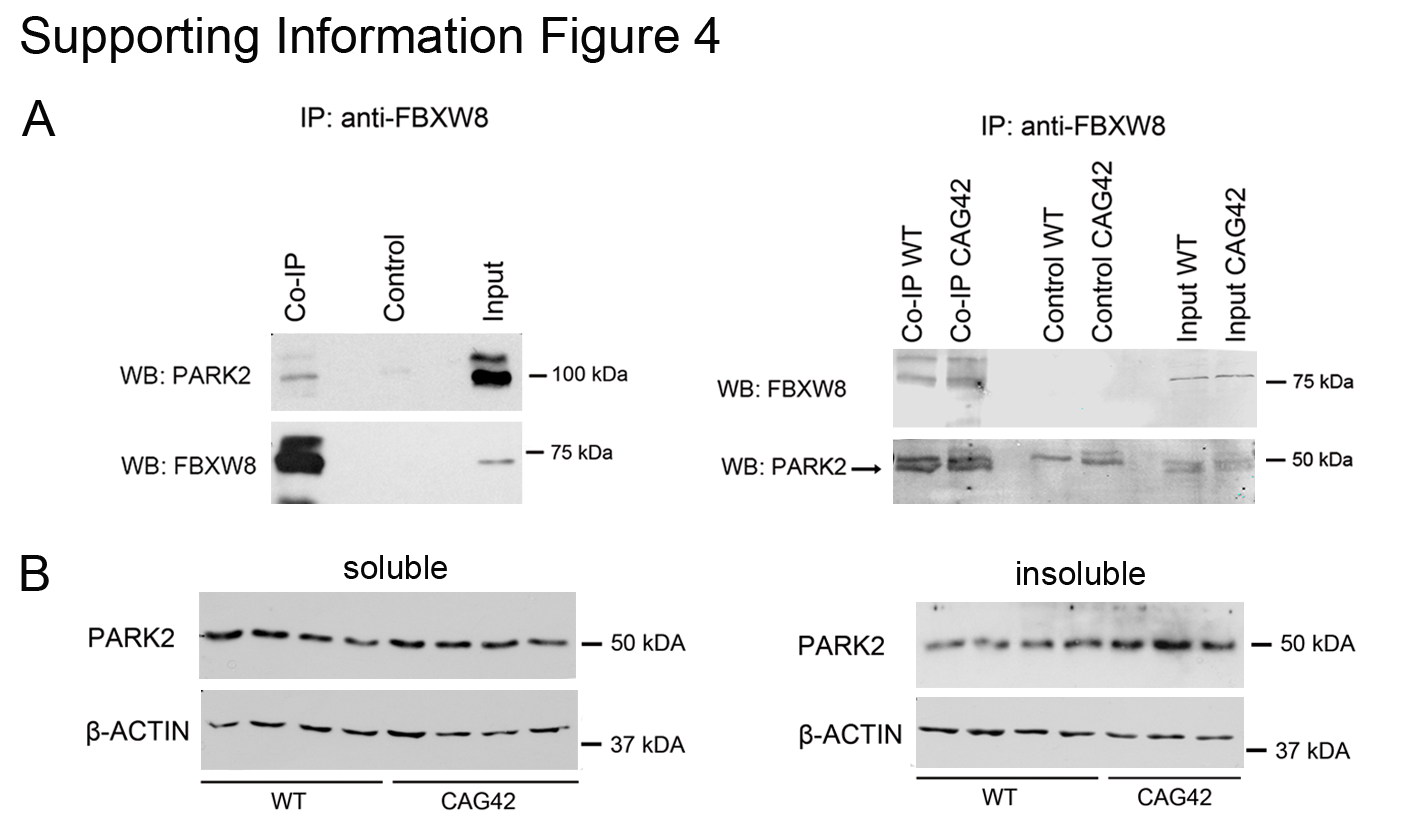

Supplement: S4 Fig — Larger cutout of Western Blot images from Fig. 3. (A) In HeLa cells overexpressing Cherry-GFP-PARK2 and FBXW8-HA, pulling with anti-FBXW8 antibody resulted in the detection of FBXW8 as well as of PARK2 in Co-IP lysates, demonstrating their interaction (experiment repeated twice, representative image). (B) PARK2 interacts with FBXW8 in Co-IP samples of Atxn2-CAG42-KIN mice independent of the polyQ length. Lower bands represent PARK2 protein (experiment repeated once). (C) PARK2 protein level is decreased in the RIPA-soluble fraction while it is increased in the SDS-soluble fraction (8 Atxn2 CAG1/CAG1 mice vs. ≥ 6 Atxn2 CAG42/CAG42 mice). (TIF) [file pone.0121089.s004.tif]

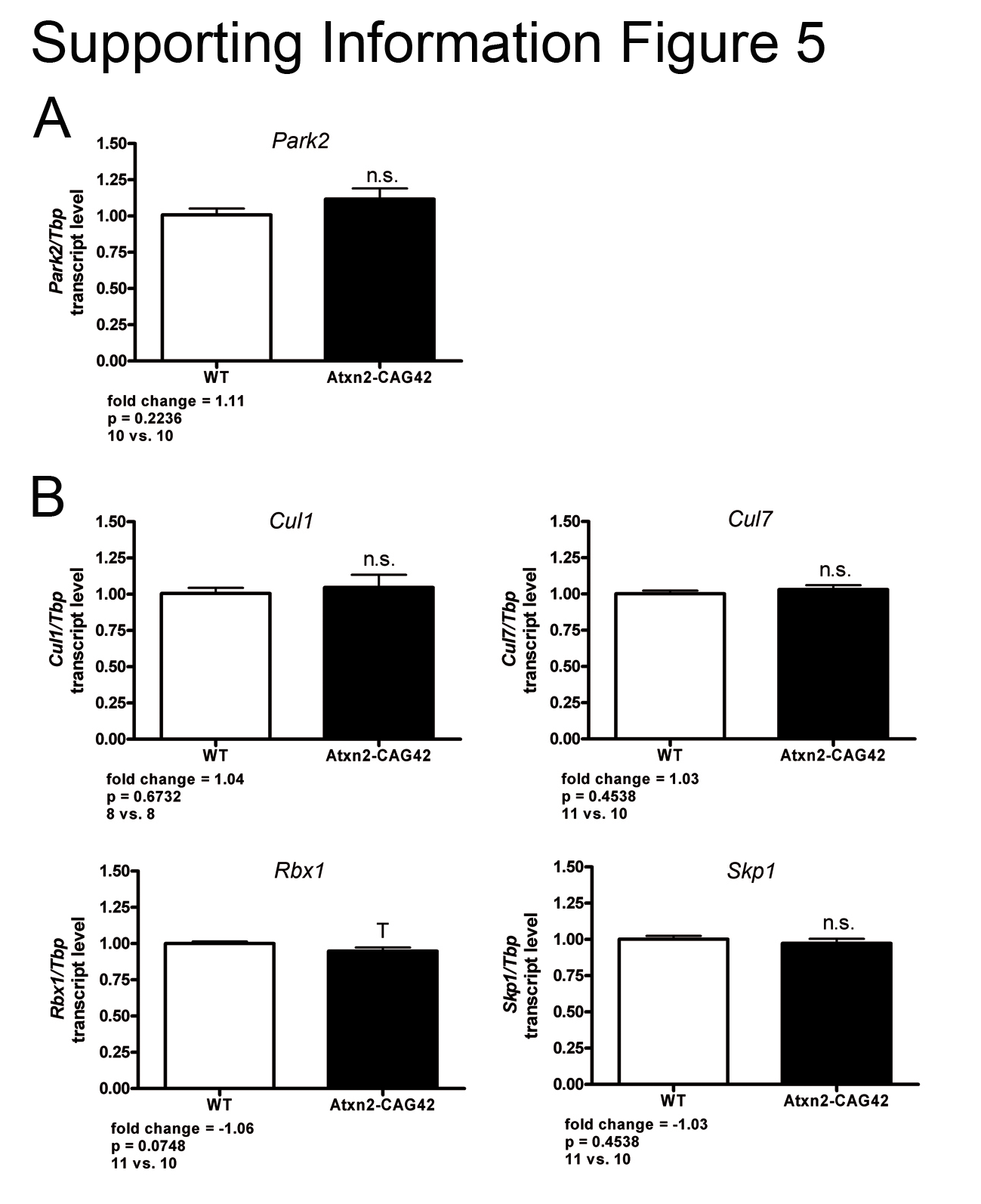

Supplement: S5 Fig — In contrast to Fbxw8, transcript levels of Cul1, Cul7, Rbx1, Skp1 (A) and Park2 (B) are not significantly changed in cerebella of 18-month-old Atxn2-CAG42-KIN mice (≥ 8 Atxn2 CAG1/CAG1 mice vs. ≥ 8 Atxn2 CAG42/CAG42 mice). n.s. = non-significant. T = trend. (TIF) [file pone.0121089.s005.tif]

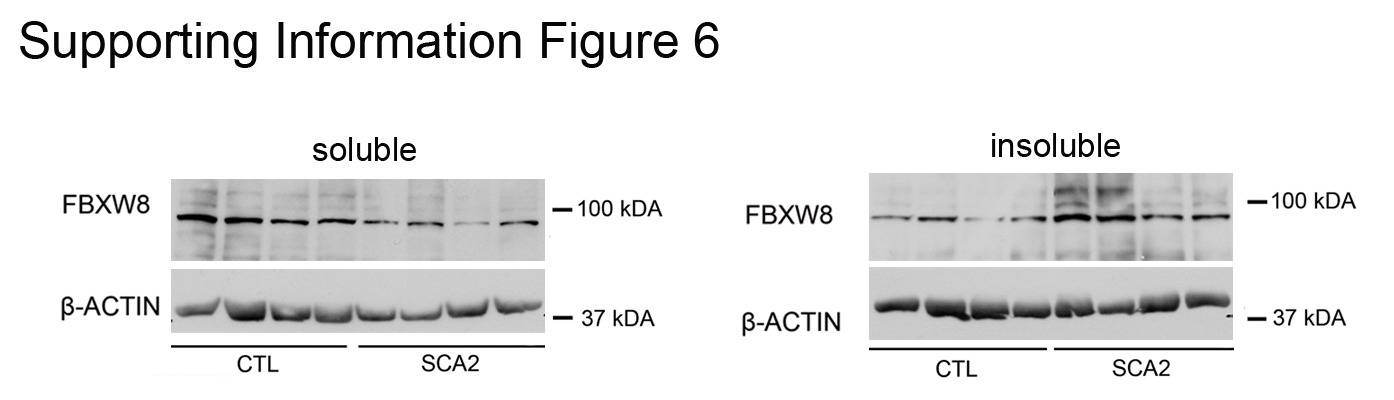

Supplement: S6 Fig — Larger cutout of Western Blot images from Fig. 4. At the protein level FBXW8 is decreased in the RIPA-soluble fraction while it is increased in the SDS-soluble fraction in SCA2 patient fibroblasts (4 CTL individuals vs. 4 SCA2 patients). (TIF) [file pone.0121089.s006.tif]
